# *Plasmodium cynomolgi* Co-infections among Symptomatic Malaria Patients, Thailand

**DOI:** 10.3201/eid2702.191660

**Published:** 2021-02

**Authors:** Chaturong Putaporntip, Napaporn Kuamsab, Urassaya Pattanawong, Surasuk Yanmanee, Sunee Seethamchai, Somchai Jongwutiwes

**Affiliations:** Chulalongkorn University Faculty of Medicine, Bangkok, Thailand (C. Putaporntip, N. Kuamsab, U. Pattanawong, S. Yanmanee, S. Jongwutiwes);; Naresuan University Faculty of Science, Pitsanulok, Thailand (S. Seethamchai)

**Keywords:** co-infections, cross-transmission, malaria, mitochondrial cytochrome oxidase I, mixed species malaria infection, parasites, PCR detection, Plasmodium, Plasmodium cynomolgi, Plasmodium falciparum, Plasmodium knowlesi, Plasmodium vivax, Thailand, vector-borne infections, zoonoses

## Abstract

Among 1,180 symptomatic malaria patients, 9 (0.76%) infected with *Plasmodium cynomolgi* were co-infected with *P. vivax* (n = 7), *P. falciparum* (n = 1), or *P. vivax* and *P. knowlesi* (n = 1). Patients were from Tak, Chanthaburi, Ubon Ratchathani, Yala, and Narathiwat Provinces, suggesting *P. cynomolgi* is widespread in this country.

*Plasmodium cynomolgi*, a simian malaria parasite, possesses biological and genetic characteristics akin to those of the most widespread human malaria parasite, *P. vivax*. Although *P. cynomolgi* circulates among monkey species such as long-tailed macaques (*Macaca fascicularis*) and pig-tailed macaques (*M. nemestrina*), experimental and accidental transmissions have been implicated in symptomatic infections in humans (*1*). Several mosquito vectors for human malaria can also transmit *P. cynomolgi*, posing the risk of cross-species transmission in areas where its natural hosts coexist with people (*1*,*2*). Among pig-tailed and long-tailed macaques living in various countries in Southeast Asia, including Thailand, *P. cynomolgi* infections are not uncommon (*3*,*4*). A case of naturally transmitted *P. cynomolgi* malaria in a human was reported from eastern Malaysia (*5*). Subsequent surveillance in western Cambodia and northern Sabah state in Malaysia revealed asymptomatic human infection, albeit at low prevalence (*6*,*7*). Symptomatic *P. cynomolgi* infection was diagnosed in a traveler returning to Denmark from Southeast Asia (*8*). During testing of symptomatic malaria patients in Thailand, we identified 9 co-infected with cryptic *P. cynomolgi* and other *Plasmodium* species. 

## The Study

We examined 1,359 blood samples taken from febrile patients who sought treatment at malaria clinics or local hospitals in 5 Thailand provinces: Tak (n = 192, during 2007–2013), Ubon Ratchathani (n = 239, during 2014–2016), Chanthaburi (n = 144, during 2009), Yala (n = 592, during 2008–2018), and Narathiwat (n = 192, during 2008–2010). Using microscopy, we found 1,152 cases in which malaria was caused by *P. vivax* (869 patients, 75.43%), *P. falciparum* (272 patients, 23.61%), or co-infection with both species (11 patients, 0.96%). Using species-specific nested PCR, including for *P. cynomolgi* (Appendix), targeting the mitochondrial cytochrome *b* gene (*mtCytb*) of 5 human malaria species for molecular detection, as described elsewhere (*9*,*10*), we found malaria in 1,180 patients; *P. vivax* infections exceeded *P. falciparum* infections (Table 1). Submicroscopic parasitemia occurred in 28/1,180 (2.4%) patients: 19 infected with *P. vivax*, 7 with *P. falciparum*, 1 with *P. vivax* and *P. falciparum*, and 1 with *P. malariae*. 

**Table 1:** Distribution of *Plasmodium* infections diagnosed by PCR of blood samples taken from febrile patients who sought treatment at malaria clinics or local hospitals in 5 provinces, Thailand*

Species	No. cases by province					Total no. cases	% Total cases
	Tak	Ubon Ratchathani	Chanthaburi	Yala	Narathiwat		
*P. vivax*	98	57	141	467	59	822	69.66
*P. falciparum*	72	41	0	87	73	273	23.14
*P. knowlesi*	0	4	0	0	0	4	0.34
*P. malariae*	0	2	0	1	0	3	0.25
*P. ovale*	0	0	0	1	0	1	0.09
*P. vivax* + *P. falciparum*	21	8	0	11	15	55	4.66
*P. vivax* + *P. knowlesi*	0	3	2	0	4	9	0.76
*P. vivax* + *P. cynomolgi*	1	1	1	3	1	7	0.59
*P. vivax* + *P. knowlesi* + *P. cynomolgi*	0	0	0	1	0	1	0.09
*P. falciparum* + *P. knowlesi*	0	3	0	1	0	4	0.34
*P. falciparum* + *P. cynomolgi*	0	0	0	1	0	1	0.09
PCR-positive	192	119	144	573	152	1,180	100.00
PCR-negative	0	120	0	19	40	179	NA
Total no. samples tested	192	239	144	592	192	1,359	NA
*NA, not applicable.							

The mean age of all patients was 26.3 (range 7–85) years; 940/1,180 (79.7%) of patients were men. Febrile symptoms, lasting 1–7 days (mean 3.1, SD ±1.3 days) before blood sample collection, developed in all PCR-positive malaria patients. Monoinfection with *P. knowlesi* occurred in 4 patients, *P. malariae* in 3, and *P. ovale* in 1. We detected co-infections in 77 (0.93%) patients; of these co-infections, 55 were *P. falciparum* and *P. vivax*. In total (i.e., including both monoinfections and co-infections), *P. knowlesi* was detected in 18 patients, of which 10 cases were newly identified from Ubon Ratchathani Province, which borders Cambodia and Laos. 

We detected *P. cynomolgi* in 9 patients, all of whom were co-infected with *P. vivax* (n = 7), *P. falciparum* (n = 1), or both *P. vivax* and *P. knowlesi* (n = 1). The overall prevalence of *P. cynomolgi* infections was 0.76%. Patients infected with *P. cynomolgi* were found in all provinces. Although 5 of these patients were from Yala Province, the proportion of *P. cynomolgi* infections among malaria cases in each malaria-endemic area (0.52%–0.87%) was comparable. 

DNA from 10 *P. knowlesi* isolates from Ubon Ratchathani Province and the 9 *P. cynomolgi* isolates were subject to nested PCR amplification spanning a 1,318-bp region of mitochondrially encoded cytochrome c oxidase I (*mtCOX1*). Direct sequencing of the purified PCR-amplified template was successfully performed from all 10 *P. knowlesi* and from 6 *P. cynomolgi* isolates. The remaining 3 *P. cynomolgi* isolates could not be further amplified due to inadequate DNA in the samples. All *mtCOX1* sequences of *P. knowlesi* from Ubon Ratchathani Province were different from one another and distinct from those from the previous case of natural human infection in Thailand (GenBank accession no. AY598141) (*11*). All 6 amplified *P. cynomolgi* isolates contained different sequences belonging to 2 clades. One was closely related to the Gombak strain (accession no. AB444129) and the remaining 5 isolates were clustered with the RO strain (accession no. AB444126) (Figure). 

**Figure:**
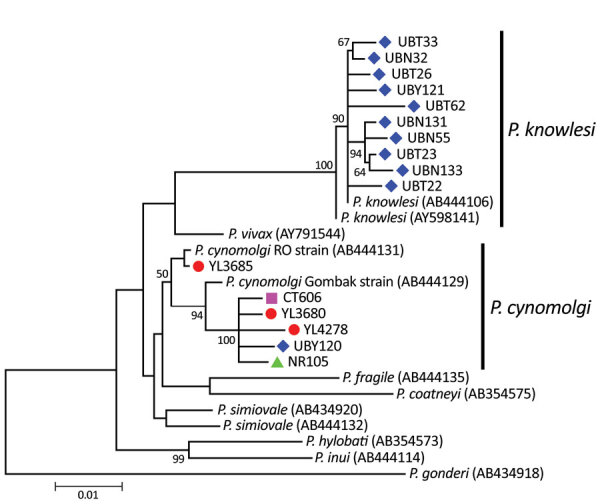
Maximum-likelihood phylogenetic tree inferred from mitochondrially encoded cytochrome *c* oxidase I of *Plasmodium cynomolgi* and *P. knowlesi* from Thailand compared with other closely related species. Tree spans 1,318-bp region. Colors indicate province where human isolates were found: red circles, Yala; green triangles, Narathiwat; purple squares, Chanthaburi; and blue diamonds, Ubon Ratchathani. GenBank accession numbers of reference sequences are given in parentheses. Bootstrap values >50% based on 1,000 pseudoreplicates are shown on the branches. Scale bar indicates nucleotide substitution per site.

All but 1 *P. cynomolgi* infection occurred in male patients (age 15–53 years, median 32 years). Most *P. cynomolgi* malaria patients resided in areas where domesticated or wild macaques were living in proximity to humans. Infections with *P. cynomolgi* occurred in different annual periods; more cases were detected in rainy seasons than in dry seasons (Table 2). The parasite density of *P. cynomolgi* could not be determined from blood smears because of morphologic resemblance to *P. vivax*; an isolate co-infected with *P. falciparum* (YL3634) had very low parasitemia. Of 8 patients with *P. cynomolgi* co-infection, 6 had parasitemia <10,000 parasites/μL (<0.2% parasitemia). It remains unknown whether *P. cynomolgi* was co-responsible for symptomatic infections or merely coexisted asymptomatically with other human malaria parasites. However, self-reported defervescence among *P. cynomolgi*–co-infected patients occurred 1–3 days after antimalarial treatment with chloroquine plus primaquine after onsite microscopic diagnosis of *P. vivax* malaria or artesunate plus mefloquine for *P. falciparum* malaria. Unfortunately, data on long-term follow-up were not available. 

**Table 2:** Demographic and parasitologic features of *Plasmodium cynomolgi*–co-infected patients among febrile patients who sought treatment at malaria clinics or local hospitals in 5 provinces, Thailand

Patient*	Age, y/sex	Province	Month	Season	Monkey in proximity	Microscopy diagnosis	Parasites/μL‡	PCR diagnosis
TSY1522	38/M	Tak	2007 Nov	Dry	No	*P. vivax*	12,160	*P. vivax,* *P. cynomolgi*
CT606†	30/M	Chanthaburi	2009 Oct	Rainy	Yes	*P. vivax*	86,535	*P. vivax,* *P. cynomolgi*
UBY120	32/M	Ubon Ratchathani	2015 Aug	Rainy	Yes	*P. vivax*	570	*P. vivax,* *P. cynomolgi*
NR105	53/M	Narathiwat	2008 Jul	Rainy	Yes	*P. vivax*	4,620	*P. vivax,* *P. cynomolgi*
YL3179	15/M	Yala	2016 Apr	Dry	Yes	*P. vivax*	1,140	*P. vivax,* *P. knowlesi* *P. cynomolgi*
YL3634	40/F	Yala	2016 Dec	Rainy	Yes	*P. falciparum*	60	*P. falciparum*, *P. cynomolgi*
YL3680	49/M	Yala	2016 Dec	Rainy	Yes	*P. vivax*	3,720	*P. vivax,* *P. cynomolgi*
YL3685	18/M	Yala	2016 Dec	Rainy	Yes	*P. vivax*	4,680	*P. vivax,* *P. cynomolgi*
YL4278	21/M	Yala	2017 Oct	Rainy	Yes	*P. vivax*	7,440	*P. vivax,* *P. cynomolgi*
*Alphanumeric designations represent provinces and serial number of blood samples. †Patient from Cambodia, but had lived in Thailand for 1 year just prior to illness, with no history of travel outside of the country. ‡All species of malaria parasites (all stages) were determined from >200 leukocytes on Giemsa-stained thick blood films.								

## Conclusions 

This report highlights the presence of *P. cynomolgi* in the human population of Thailand, where natural hosts, both pig-tailed and long-tailed macaques, are prevalent. All patients with *P. cynomolgi* infections harbored either *P. falciparum* or *P. vivax* in their blood, implying that this simian malaria species could share the same anopheline vectors or have different vectors with similar anthropophilic and zoophilic tendencies. The presence of *P. cynomolgi* in diverse malaria-endemic areas of Thailand suggests that cross-species transmission has occurred. Human infection with *P. cynomolgi* seems not to be newly emerging because it was detected among blood samples collected over a range of time periods since 2007. Undoubtedly, morphologic similarity between *P. cynomolgi* and *P. vivax* can hamper conventional microscopic diagnosis (*1*,*5*,*8*). Cryptic co-existence of simian and human malaria species could further preclude accurate molecular detection when inadequate diagnostic devices are used. 

Previous surveys of *Plasmodium* infections in pig-tailed and long-tailed macaques have revealed the presence of *P. cynomolgi* and other simian malaria species in Thailand, mainly in the southern part of the country (*4*). Most patients infected with *P. cynomolgi* resided in areas where macaques were living in proximity to humans; therefore, the risk of acquiring malaria from this parasite could increase as people encroach into the habitats of infected macaques, as happened with malaria caused by *P. knowlesi*. Of note, co-infection with *P. cynomolgi*, *P. knowlesi*, and *P. vivax* occurred in a patient in Yala Province whose housing area was surrounded by several domesticated pig-tailed and long-tailed macaques.

Analysis of the *mtCOX1* sequences of *P. cynomolgi* among 6 patients showed that all isolates possessed different genetic sequences, suggesting that several strains or clones of this simian parasite are capable of cross-transmission from macaques to humans. Meanwhile, *P. cynomolgi* seems to contain 2 divergent lineages (*12*), represented by RO and Gombak strains. The *mtCOX1* sequences of both *P. cynomolgi* lineages were found in human-derived isolates in this study, further supporting that diverse strains of this parasite can infect people. Likewise, sequence diversity in the *mtCOX1* of *P. knowlesi* from Ubon Ratchathani Province suggests that cross-transmission from macaques to humans may not be restricted to particular parasite strains. 

Although human malaria from either parasite may be asymptomatic, infection with *P. knowlesi* can result in death, but patients infected with *P. cynomolgi* at worst had only benign symptoms (*5*–*8*). However, severe and complicated malaria has been observed in rhesus macaques experimentally infected with *P. cynomolgi* (*13*). 

Whether severe cynomolgi malaria can occur in humans remains to be elucidated. However, if human infections with *P. cynomolgi* do become public health problems, diagnostic and control measures might be complicated by the morphological similarity between *P. vivax* and *P. cynomolgi*. This possibility makes further surveillance of this simian malaria in humans mandatory. 

Appendix Additional methodology for *Plasmodium cynomolgi* co-infections among symptomatic malaria patients, Thailand.
