# Supplementary material for: Plasmodium cynomolgi Co-infections among Symptomatic Malaria Patients, Thailand
Source: Emerg Infect Dis. 2021 Feb;27(2):590–3. doi: 10.3201/eid2702.191660 (PMC7853550; doi:10.3201/eid2702.191660)
Supplement: Appendix — Additional methodology for Plasmodium cynomolgi co-infections among symptomatic malaria patients, Thailand. [file 19-1660-Techapp-s1.pdf]

Article DOI: https://doi.org/10.3201/eid2702.191660

Plasmodium cynomolgi Co-infections among Symptomatic Malaria Patients, Thailand

Appendix

We performed primary DNA amplification in a 30 µL reaction mixture containing template DNA, 2.5 mmol/L $MgCl_2$, 300 mmol/L each deoxynucleoside triphosphate, 3 µL of 10X ExTaq PCR buffer (Takara Bio; https://www.takara-bio.com), 0.3 µmol/L of primers PCOX1-F0 and PCOX1-R0, and 1.25 units of ExTaq DNA polymerase (Takara Bio). The thermal cycle profile contained preamplification denaturation at 94°C for 1 min followed by 35 cycles of 94°C for 40 s; 50°C for 30 s, 72°C for 1 min, and a final extension at 72°C for 5 min. We performed secondary PCR in 2 separate reactions in a total volume of 30 µL, one using primers Pcy1COX1-F and PcyCOX1-R and the other using primers Pcy2COX1-F and PcyCOX1-R. The reaction mixtures were essentially the same as those for primary PCR except for the primers and 2 µL of primary PCR product as templates. We performed all amplifications in an Applied Biosystem GeneAmp PCR System 9700 thermocycler (PE Biosystems, https://www.thermofisher.com) and analyzed the DNA fragments using 2% agarose gel electrophoresis. The expected PCR fragments from primary PCR were 1,481 bp and from secondary PCR were 317–320 bp.

Appendix Table 1. Nested PCR detection of *Plasmodium cynomolgi*

Primers	Sequence (5′ → 3′)	Positions after the RO strain*
	Primary PCR	
PCOX1-F0	CTTTTAACGCCTGACATGGATGGATAATACTCG	3,196–3,228
PCOX1-R0	TCTGGATAATCAGGAATACGTCTAGGCATTAC	4,645–4,676
	Secondary PCR	
Pcy1COXI-F	CCAAGCCTCACTTATTGTTAATTTATTTTT	3,291–3,320
Pcy2COXI-F	CTTATTGTTAATTATATATTGTATTATATATTTTTTG	†
PcyCOXI-R	CTGGAGAACCACATAAAATTGGTAAAAAA	3,579–3,607

*GenBank accession number AB444131
†Sequence after *P. cynomolgi* from macaques in Thailand not found in the sequence of the RO strain (Putaporntip et al., unpub. data)

Appendix Table 2. Pairwise sequence comparison of the mitochondrial cytochrome oxidase I genes of human and simian malaria species*†

Strain	*P. cynomolgi* RO strain AB444131	*P. cynomolgi* Gombak strain AB444129	*P. fieldi* AB444132	*P. simiovale* AB434920	*P. vivax* AY791544	*P. fragile* AB444135	*P. knowlesi* AY598141	*P. hylobati* AB354573	*P. coatneyi* AB354575	*P. inui* AB444114	*P. gonderi* AB434918	*P. malariae* AB354570	*P. ovale* AB354571	*P. falciparum* AJ276845
P. cynomolgi RO strain AB444131	NA	12	15	16	21	39	47	54	55	62	80	105	103	172
P. cynomolgi Gombak strain AB444129	99.09	NA	16	19	21	36	33	51	49	36	83	112	107	180
P. fieldi AB444132	98.79	98.86	NA	16	23	35	37	42	47	57	80	116	110	184
P. simiovale AB434920	98.79	98.56	98.79	NA	19	38	34	43	45	58	78	112	110	182
P. vivax AY791544	98.56	98.25	98.41	98.41	NA	31	39	42	44	44	76	106	114	180
P. fragile AB444135	97.65	97.12	97.34	97.27	97.04	NA	33	38	40	44	77	109	113	179
P. knowlesi AY598141	97.50	97.04	97.42	97.19	97.50	96.43	NA	34	40	45	75	104	112	180
P. hylobati AB354573	97.42	97.12	96.81	96.74	96.81	96.13	95.90	NA	38	43	65	109	108	180
P. coatneyi AB354575	97.12	96.97	96.97	96.66	96.59	96.43	96.28	95.83	NA	38	66	102	104	176
P. inui AB444114	97.12	96.74	96.59	96.66	96.66	95.60	95.68	97.27	95.30	NA	64	106	96	174
P. gonderi AB434918	95.14	94.99	95.07	94.31	94.16	94.23	94.08	93.93	93.70	93.93	NA	103	101	177
P. malariae AB354570	92.19	91.96	92.26	91.73	92.11	91.73	91.96	91.50	91.20	91.50	92.03	NA	104	174
P. ovale AB354571	92.11	92.34	92.72	92.11	91.81	91.50	91.43	91.35	91.65	91.65	91.88	92.19	NA	176
P. falciparum AJ276845	86.65	86.80	86.57	86.80	86.65	86.34	86.34	86.42	86.34	86.19	86.04	86.34	86.95	NA

*NA, not applicable.
†Homology inferred from percentage sequence identity are shown in the lower left corner cells and the numbers of pairwise nucleotide differences in the upper right corner cells. Sequences contain 1,318 bp. GenBank accession numbers are shown below the species names.
